# Foliar Silicon Application at Multiple Growth Stages Can Improve Lodging Resistance, Yield, and Grain Quality in Rice

**DOI:** 10.3390/plants15142133

**Published:** 2026-07-10

**Authors:** Ruilin Guo, Tianyu Du, Jianghui Yu, Ying Zhu, Guodong Liu, Fangfu Xu, Qun Hu, Haiyan Wei, Guangyan Li

**Affiliations:** 1 Jiangsu Key Laboratory of Crop Genetics and Physiology/Jiangsu Key Laboratory of Crop Cultivation and Physiology, Agricultural College, Yangzhou University, Yangzhou 225009, China; 2 Jiangsu Co-Innovation Center for Modern Production Technology of Grain Crops, Yangzhou University, Yangzhou 225009, China; 3 Research Institute of Rice Industrial Engineering Technology, Yangzhou University, Yangzhou 225009, China

**Keywords:** silicon, japonica rice, yield, rice quality, lodging resistance, nitrogen use efficiency

## Abstract

Silicon is an essential element for enhancing the yield, quality and lodging resistance of rice, which are mainly applied through a base application or foliar application during critical growth stages in current practice. The effect of foliar application of water-soluble silicon fertilizer at different growth stages on rice yield, quality and lodging resistance were studied to screen the optimal spraying period that enhances both high yield and lodging resistance. The experiment used “Nanjing9108 and Nanjing5718” as test materials; three treatment combinations were established, corresponding to the following spraying periods: tillering stage + elongation stage (Si1), tillering stage + elongation stage + grain-filling stage (Si2), elongation stage + grain-filling stage (Si3). Applying with plain water served as the control (CK). The results showed that a foliar application of silicon fertilizer significantly increased rice yields, with the Si2 treatment yielding the best results, increasing the yields of the NJ9108 and NJ5718 by 4.82–5.70% and 4.02–5.29%, respectively, over the two-year period. Foliar silicon fertilization significantly enhanced stem wall thickness and shortened the length of the basal internodes, thereby enhancing the lodging resistance of rice. The lodging resistance of the second basal internodes by 5.52–7.79% for NJ9108 and 4.80–7.15% by NJ5718, while that of the third basal internodes increased by 3.42–4.97% and 5.67–6.76%, respectively. In addition, silicon application treatments exhibited enhanced N uptake at maturity with increases of 6.19–13.56% and 5.37–13.86%, respectively. N use efficiency increased with the number of silicon applications. Silicon application during the grain-filling stage effectively delayed leaf senescence, providing sustained photosynthesis to support dry matter accumulation during the mid-to-late growth stages and further facilitating nitrogen transport and utilization. In summary, the synergistic application of silicon fertilizer during the tillering, elongation, and grain-filling stages is the most effective strategy for achieving the dual objectives of high yield and lodging resistance.

## 1. Introduction

Rice, as the staple crop for more than half of the world’s population, has high and stable yields that are directly linked to national food security and sustainable agricultural development [1]. With the continuous growth of China’s population and rising living standards, there is not only a demand for stable rice production but also increasing demand for higher-quality rice. However, under large-scale and intensive cultivation methods, rice still faces many challenges. To pursue high yield, excessive nitrogen fertilizer is applied in the field, leading to increased plant density and slender stems, a higher risk of lodging in rice during the later growth stages, and low nitrogen use efficiency. In particular, against the backdrop of frequent extreme weather events, the risk of rice lodging has increased significantly, posing serious challenges to production [2]. After lodging occurs in plants, the vascular bundles of stems are compressed or fractured, which severely impedes the upward transport of water and photosynthates. The poor ventilation and light transmission conditions within the plant population lead to a decline in leaf photosynthetic capacity, resulting in uneven grain filling and reduced yield [3,4,5].

Rice is a silicon-loving crop with a high demand for silicon. Silicon is one of the most essential nutrients for rice after nitrogen, phosphorus and potassium, and it is known as the “fourth major nutrient element” for rice [6,7]. Silicon is an essential element for rice growth and development, and it is primarily absorbed by rice in the form of monosilicic acid [8,9]. It is also the only form of silicon that can be directly absorbed by plants. Silicon fertilizer exerts significant effects on improving rice yield, quality and stress resistance. Research findings indicate that silicon fertilizer can enhance the lodging resistance of plants by shortening the internode length of the stem base and increasing stem thickness [10]. In terms of disease resistance, silicon can form a physical barrier in plant cells to inhibit the invasion of fungal hyphae, thereby enhancing the resistance of plants to pathogenic bacteria [11]. At the same time, silicon effectively alleviates drought stress by promoting root water absorption, reducing transpiration rate, and enhancing photosynthesis [12]. Silicon fertilizer effectively alleviates cadmium toxicity in plants by reducing cadmium absorption and translocation, regulating the plant antioxidant system [13]. In addition to playing a positive role in rice stress tolerance, silicon can also improve rice nitrogen use efficiency. Wei et al. [14] indicated that a foliar application of silicon fertilizer promotes the nitrogen absorption capacity of rice, improves nitrogen assimilation efficiency by regulating the activities of synthetases involved in nitrogen metabolism (such as NR, GS), and ultimately increases nitrogen fertilizer use efficiency. Within a certain application range, silicon fertilizer can offset the rice demand for exogenous nitrogen, thus significantly improving nitrogen use efficiency. In addition, silicon application can enhance the activities of enzymes for grain starch synthesis, including ADP-glucose pyrophosphorylase (AGPase), soluble starch synthase (SSS) and starch-branching enzyme (SBE), to accelerate the starch synthesis rate, thereby promoting grain filling, reducing the incidence of empty and shrunken grains, increasing grain weight while decreasing the protein and amylose content in grains, and ultimately improving both the yield and quality of rice [15].

According to the form and method of application, silicon fertilizers are primarily used as solid base fertilizers and water-soluble foliar silicon fertilizers. At present, silicon mainly exists in the form of metasilicic acid in agricultural production. But metasilicic acid must be converted to monosilicic acid in the soil before it can be absorbed and utilized by rice. When applied as a basal fertilizer, silicon is generally incorporated into soil in a single application prior to plowing. This approach has the advantage of long fertilizing duration, as one single application can meet the silicon demand for two to three consecutive rice growing seasons, while its disadvantage lies in low absorption and utilization efficiency. Foliar application can be carried out during critical stages of crop growth; its advantages include low application rates, rapid absorption, and high utilization efficiency. The foliar application of silicon fertilizer often requires multiple applications to achieve significant results. The primary reason may be that silicon has poor mobility within the plant, cannot be re-transported once it accumulates in cell walls or intercellular spaces, and is difficult to transfer to other parts of the plant once absorbed by the leaves [16]. Moreover, the demand for silicon varies at different growth stages: applying silicon fertilizer during the tillering stage promotes tiller development, the period from elongation to heading is a critical period for lodging resistance, and from heading to grain filling, silicon is directly transported to the panicles, enhancing grain filling and improving grain quality [17,18]. Therefore, applying silicon fertilizer at a single stage may not meet the crop’s requirements throughout its entire growth cycle. Gong et al. [19] proposed that applying silicon fertilizer at different growth stages significantly increased yield: the best results were achieved by applying base silicon fertilizer and top dressing during the critical period of effective tillering, and the next was top dressing at the elongation stage, which increased yield by 19.54% compared to no silicon fertilizer application. Dorairaj et al. [20] proposed that applying silicon fertilizer during the reproductive growth stage can significantly improve the agronomic traits and enhance stem bending resistance. Supplemental foliar silicon fertilizer is far more effective than a single basal application or a single foliar application, providing sustained nutrient supply to meet rice growth requirements. Currently, numerous studies have been conducted both domestically and internationally on the effects of silicon on rice growth, yield, and quality. However, most research has focused on basal application or single foliar application at critical growth stages. There remains insufficient investigation into how combined foliar applications of water-soluble silicon fertilizer at different growth stages affect rice yield, quality, and lodging resistance. Therefore, the objective was to explore through field trials the impact of combined foliar silicon applications at various growth stages on rice yield, quality, and lodging resistance and thereby identify the optimal combination of application periods for foliar silicon fertilizer, providing scientific theoretical support and technical guidance for a green, high-yield, and high-quality cultivation of premium japonica rice.

## 2. Materials and Methods

### 2.1. Experimental Location and Test Variety

Field experiments were conducted at Chenxing Village (32°31′ N, 119°55′ E), Yangzhou City, in 2024 and 2025. The previous crop in the experimental field was wheat with sandy loam soil and balanced soil fertility. The soil organic matter concentration was 31.72 g/kg, the alkali-hydrolyzable N concentration was 221.1 mg/kg, the available P concentration was 33 mg/kg, and the available K concentration was 210 mg/kg. The experimental variety was late-maturing medium japonica rice Nanjing9108 and Nanjing 5718. Its whole growth duration is about 155 days and 150 days, respectively. NJ9108 has a relatively slender stem, and its lodging resistance is weak, while NJ5718 has a thick and sturdy stem, and it shows resistance to lodging. Taking the above into account, the two varieties were selected as the experimental materials. 

### 2.2. Experimental Design

The experiments were sown on 20 May 2024 and 19 May 2025, respectively, with transplanting carried out on 15 June. The plant spacing was 12 × 30 cm with 4–5 seedlings per hill. Each plot was 35 m^2^ with three replications. The foliar spraying concentration was all 5 g/L (water-soluble silicon fertilizer provided by Anhui Leyouyou Agricultural Science and Technology Co., Ltd. Wuhu, China, the main component is monosilicic acid in solution, with technical index Si ≥ 100 g/L). Four treatments were set: CK: spraying an equal amount of clean water as control; Si1: applying silicon fertilizer during the tillering and the elongation stage; Si2: applying silicon fertilizer during tillering, elongation and grain-filling stages; and Si3: applying silicon fertilizer during elongation and grain-filling stages. The specific experimental designs are shown in Table 1. The N fertilizer in the experiment was 270 kg hm^−2^ and was applied with basal, tillering, and panicle fertilizer at a ratio of 3.5:3.5:3 at the relevant growth stage. The ratio of nitrogen (N): phosphorus (P_2_O_5_): potassium (K_2_O) was 2:1:2 [21]. Cultivation practices related to water management and the control of pests, diseases, and weeds were implemented in accordance with high-yield cultivation requirements.

### 2.3. Sampling and Data Collection

#### 2.3.1. Yield and Yield Components

At maturity, 50 random hills per treatment were sampled for effective panicle number. Five of them were used to determine the grains per panicle, seed-setting rate, and 1000-grain weight. Yield was measured from 100 hills after threshing, impurity removal, and air drying, and it was adjusted to 14.5% moisture.

#### 2.3.2. Leaf Area Index (LAI)

At the elongation, heading, and maturity stages, three hills per plot were sampled. The leaf area was measured with a portable leaf area meter (Li-3000A, LI-COR Biosciences, Lincoln, NE, USA).

#### 2.3.3. Dry Matter Accumulation

At the elongation, heading, and maturity stages, six hills per plot were sampled, among which two hills were used as whole samples, and four holes were divided into organs (leaves and stems before heading; leaves, stems, and panicles after heading). Samples were oven-killed (105 °C, 30 min) then dried (80 °C to constant weight) to determine the dry weight of whole plants and organs.

#### 2.3.4. Rice Quality

The rice grains were naturally air-dried, stored at room temperature for 90 days, and then air-selected with a winnowing machine to determine the rice grain quality. A 150 g sample of rice grains was passed through a de-husker for polishing and then separated into broken and unbroken grains. The brown rice rate, milled rice rate, and head rice rate were expressed as percentages of the total (150 g) rice grains. The values for the appearance quality (chalky grain percentage and chalkiness degree) were calculated using a rice appearance quality detector (Hangzhou Wanshen Detection Technology Co., Ltd., Hangzhou, China). The rice taste meter (Satake Corporation, Higashi-Hiroshima, Japan) was used to automatically measure the taste value of rice. The protein and amylose content of milling rice were determined using a 1241 NIR rapid quality analyzer (Infrared 1241 grain analyzer) manufactured by Foss Tecator, Hillerod, Denmark [22].

#### 2.3.5. Stem Morphology and Mechanical Properties

At 10 days after heading, three representative plants were randomly taken from each plot, and five representative stems with uniform growth were selected. The bending resistance of the second and third internodes from the base was measured using a plant stem strength meter (model TP-YYD-1, Zhejiang Topu Yunnong Technology Co., Ltd., Hangzhou, China) [23]. At the same time, the basal internode length, internode diameter, and stem wall thickness were measured using a ruler and a vernier caliper.

#### 2.3.6. N Content

Plant and organ samples (above stages) were ground and sieved (80-mesh). The nitrogen concentration was determined by the Kjeldahl method, and the total nitrogen content was measured using an automatic Kjeldahl analyzer (Kjeltec 8400, Foss Tecator, Hillerod, Denmark) [24] with N use efficiency-related indices calculated simultaneously.

#### 2.3.7. Data Calculation and Statistical Methods

Microsoft Excel 2019 and SPSS 26.0 were used for data processing and correlation analysis. The LSD test was applied at a 0.05 probability level to establish the significance of discrepancy among each mean.

Nitrogen accumulation (NA) in rice refers to the total amount of nitrogen taken up by the root system and accumulated in the aboveground plant parts (stems, leaves, and panicles) at a given growth stage or over the entire growing season. The formula is as follows:

N absorption (NA, kg hm^−2^) = aboveground dry weight at that stage × N content;

NAPE reflects the proportion of applied nitrogen fertilizer absorbed by the crop. The formula is as follows:

N apparent efficiency (NAPE, %) = (total N accumulation of plants in N-applied plots − total N accumulation of plants in N free plot)/N application rate × 100;

NPE reflects the efficiency with which absorbed nitrogen is converted into grain yield. The formula is as follows:

N physiological efficiency (NPE, kg kg^−1^) = (rice yield in N-applied plots − rice yield in N free plot)/(total N accumulation of plants in N-applied plots − total N accumulation of plants in N free plot);

NAGE reflects the grain yield increase per unit of nitrogen fertilizer applied. The formula is as follows:

N agronomic efficiency (NAGE, kg kg^−1^) = (rice yield in N-applied plots − rice yield in N free plot)/N application rate;

NPFP reflects the total grain yield per unit of nitrogen fertilizer input. The formula is as follows:

N partial factor productivity (PFP, kg kg^−1^) = grain yield/N application rate;

## 3. Results

### 3.1. Yield and Its Components

Multiple foliar sprays of silicon fertilizer at different growth stages had varying effects on rice yield and yield components. Applying (Si2) once each during the tillering, stem elongation, and grain-filling stages resulted in the greatest yield increase (Table 2). For NJ9108, the yield reached 9.46 t hm^−2^ and 9.79 t hm^−2^, which were significant increases of 5.70% and 4.82% compared with CK, respectively. For NJ5718, yield reached 10.54 t hm^−2^ and 10.87 t hm^−2^, representing significant increases of 5.29% and 4.02%, respectively. The differences were significant compared to other foliar fertilization treatments. The Si3 and Si1 treatments yield increased of 2.874.02% and 1.93–2.68% compared with CK, respectively.

In terms of the yield components, the two varieties showed basically consistent trends over the two years. Both the number of effective panicles and the number of spikelets per panicle decreased as the timing of silicon fertilizer application was delayed. Spraying silicon fertilizer during the tillering and elongation stages had the most significant effect on increasing the number of panicles and spikelets per panicle in rice. Based on the combined results from the two years, the number of panicles in NJ9108 and NJ5718 increased by 2.86–3.37% and 1.81–2.89%, respectively, the number of spikelets per panicle increased by 2.01–3.43% and 3.59–3.60%, respectively; the application of silicon fertilizer at different stages had little effect on the 1000-grain weight and seed-setting rate.

### 3.2. Leaf Area Index and Tiller Dynamics

As shown in Table 3, the application of silicon fertilizer (Si2) during the tillering, elongation and maturity stages significantly increased the leaf area index, while its application during the grain-filling stage effectively alleviated leaf senescence. Taking the two-year results together, compared with CK, the Si2 increased the leaf area index of NJ9108 and NJ5718 by 4.54–15.32% and 4.64–17.28% at the heading stage and maturity stage, respectively. Compared with Si1, the leaf area index of NJ9108 and NJ5718 under the Si2 treatment at the maturity stage increased by 4.09–5.70% and 4.64–4.80%, respectively, while that of the two varieties under the Si3 treatment increased by 0.78–1.02% and 1.90–1.96%, respectively.

Foliar application of silicon fertilizer increased the number of tillers, especially Si2 treatment. The number of tillers in the Si1 and Si2 treatments was significantly higher than that in the CK and Si3 treatments at the elongation stage and heading stage (Table 4). Compared with CK, the number of tillers in the NJ9108 and NJ5718 under Si1–Si3 at maturity was 1.79–9.99% and 1.50–5.16% higher, respectively. For the Si2 treatment, the percentage of productive tillers in both varieties was 2.79% and 3.35% higher than CK, respectively.

### 3.3. Dry Matter Accumulation

As shown in Table 5, the application of silicon fertilizer during the tillering stage (Si1, Si2) significantly increased the dry matter accumulation of rice before the elongation stage. Two applications during the tillering and elongation stages (Si1, Si2) significantly increased the population dry matter accumulation at the heading stage. Compared with a single application during the elongation stage (Si3), NJ9108 and NJ5718 showed higher increases of 1.73–2.12% and 1.46–1.95%, respectively. Dry matter accumulation at the maturity stage was highest in the treatment with three applications (Si2), which was followed by the Si3 treatment with two applications during the elongation and grain-filling stages. Compared with CK, the dry matter accumulation of the two varieties treated with Si2 increased by 5.15% and 3.26% on average at the maturity stage, while the Si3 treatment showed an average increase of 4.15% and 2.74%. The results from the two-year trials were generally consistent for both varieties.

### 3.4. Processing and Appearance Quality

The foliar application of silicon fertilizer improved the processing and appearance quality of rice (Table 6). Over the two-year experiments, the trends in the brown rice rate, milling rice rate, and head milling rice rate followed the trend Si2 > Si3 > Si1 > CK. Over the two-year period, the head milling rate of treatments Si1, Si2 and Si3 of NJ9108 increased by 0.88%, 1.55% and 1.36% on average, respectively; compared with CK, the average head milling rate of treatments Si1, Si2 and Si3 of NJ5718 increased by 0.99%, 1.51% and 1.26%, respectively. Further comparison of the effects of silicon fertilizer on the appearance quality of rice revealed that the foliar application of silicon fertilizer improved the chalky grain percentage and chalkiness degree of rice to a certain extent, but the differences among various treatments were minor. In the two-year experiments, the Si2 treatment consistently showed the lowest rice chalkiness degree of rice.

### 3.5. Nutritional and Taste Quality

The protein content and amylose content of rice decreased with delay of the application period of silicon fertilizer (Table 7). The protein content of NJ9108 and NJ5718 in the Si2–Si3 treatments decreased significantly by 3.67–5.56% and 5.60–6.88% compared with CK; compared with the Si1 treatment, the protein content of the two varieties in the Si2–Si3 treatments decreased by 1.66–3.55% and 2.61–3.63%, respectively. The amylose content of NJ9108 and NJ5718 in the Si2–Si3 treatments decreased significantly by 2.83–5.34% and 2.38–4.69% compared with CK. Compared with the Si1 treatment, the amylose content of the two varieties in the Si2–Si3 treatments decreased by 2.07–4.32% and 1.35–3.51%, respectively.

Compared with CK, the foliar application of silicon fertilizer in 2024 increased the taste values of NJ9108 and NJ5718 by 2.79–5.88% and 1.21–1.74%, respectively. In 2025, the taste values of the Si1-Si3 treatments for NJ9108 and NJ5718 increased by 2.77–5.84% and 1.75–2.11%, respectively, with the Si2 and Si3 treatments yielding the highest taste values.

### 3.6. Plant Architecture

**Figure 1:**
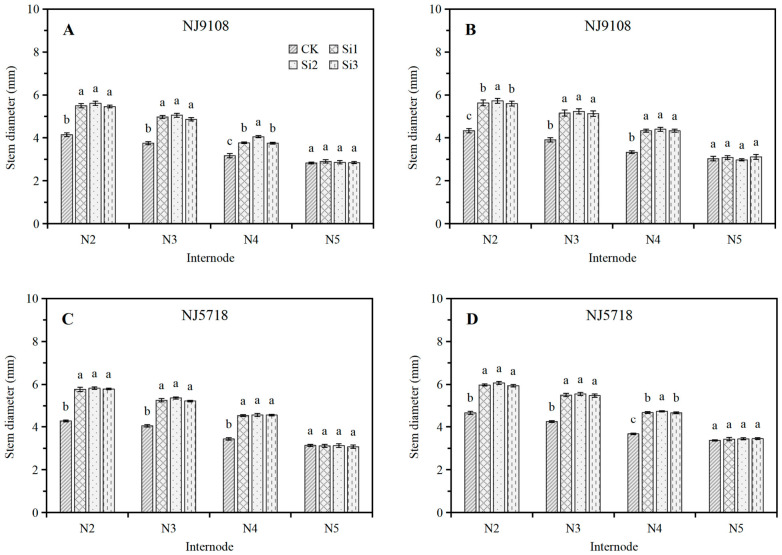
Effect of foliar silicon fertilizer on stem diameter. (**A**,**C**): 2024; (**B**,**D**): 2025. N2: the second basal internode. N3: the third basal internode. N4: the fourth basel internode. N5: the fifth basel internode. The same convention applies to subsequent figures. Different lowercase letters following data within the same column indicate significant differences among treatments at the 5% probability level (*p* < 0.05). The same convention applies to subsequent tables.

**Figure 2:**
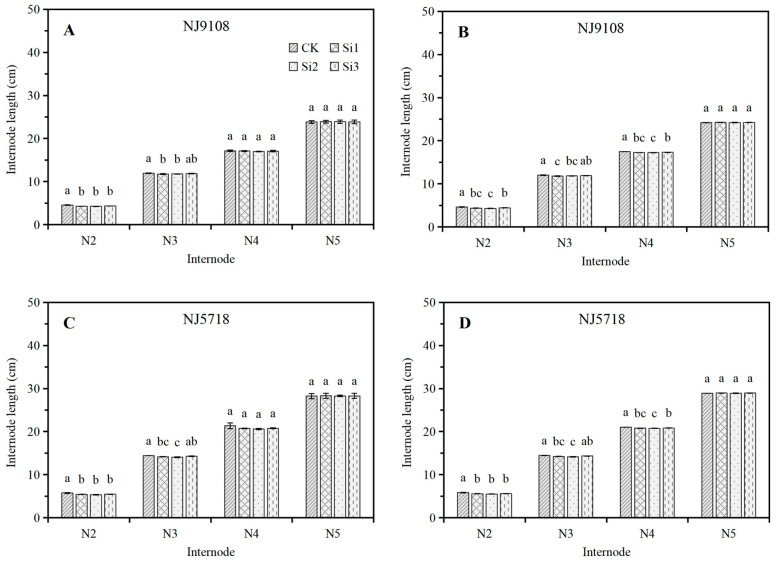
Effect of foliar silicon fertilizer on internode length. (**A**,**C**): 2024; (**B**,**D**): 2025. Different lowercase letters following data within the same column indicate significant differences among treatments at the 5% probability level (*p* < 0.05). The same convention applies to subsequent tables.

The application of foliar silicon fertilizer significantly increased the stem diameter at the lower internodes of rice plants, but it had no significant effect on the upper internodes (N3, N4) (Figure 1). Based on two-year trials, compared with CK, the stem diameters of N2 and N3 internodes in NJ9108 treated with silicon fertilizer increased by 30.30–33.49% and 32.13–34.89%, respectively, while those in NJ5718 increased by 30.88–32.73% and 29.04–31.59%. By comparing the same internodes, silicon application reduced the length of lower internodes to some extent (Figure 2) with the Si2 treatment showed the shortest internode lengths. In NJ9108, the average lengths of N2 and N3 internodes under Si2 treatment were reduced by 7.15% and 1.46% compared with CK, respectively, while in NJ5718, they were reduced by 6.83% and 2.39%.

### 3.7. Bending Resistance and Stem Wall Thickness

The results showed that the foliar application of silicon fertilizer significantly increased the bending resistance of the second and third internodes at the base (Table 7). Compared with CK, the average bending resistance of the N2 and N3 internodes in NJ9108 increased by 5.52–7.79% and 4.80–7.15%, respectively, while in NJ5718, they increased by 3.42–4.97% and 5.67–6.76%. In 2024, the second internode of NJ9108 and NJ5718 increased by 6.25% and 7.95% compared with CK, respectively. In 2025, the second internode of NJ9108 and NJ5718 increased by 6.47% and 4.59%, respectively. Across both years, the Si2 treatment yielded the best results for both bending resistance and stem wall thickness in both varieties with only minor differences among different silicon fertilizer treatments, indicating that applying silicon fertilizer at the elongation stage is a critical period for enhancing rice bending resistance. Comparing Si1 and Si3 revealed that applying silicon fertilizer at the tillering stage was more effective than at the grain-filling stage in improving stem bending resistance (Table 8).

### 3.8. N Accumulation

As shown in Table 9, the foliar application of silicon fertilizer significantly increased nitrogen accumulation in the aboveground parts of rice plants. Applying silicon fertilizer during the tillering stage increased nitrogen accumulation before the elongation stage. Applying silicon fertilizer during the tillering and elongation stages increased the nitrogen accumulation in rice plants at the heading stage, and two applications (Si1 and Si2) were significantly higher than the single application at the elongation stage (Si3): the nitrogen accumulation of NJ9108 and NJ5718 increased by 6.29–8.89% and 9.10–10.72%, respectively. The nitrogen accumulation in the maturity stage was highest with Si2 treatment, which was followed by the application during the elongation and grain-filling stages (Si3). The results from the two-year trials were generally consistent for both varieties.

### 3.9. N Use Efficiency

With the increase in silicon fertilizer application frequency, NAPE, NAGE and PFP increased correspondingly, and the Si2 treatment performed the best (Table 10). In 2024, compared with CK, NJ9108 Si2 treatment increased NAPE, NAGE, and PFP by 35.64%, 22.54%, and 5.67%, respectively, while NJ5718 increased by 42.15%, 23.83%, and 5.34%, respectively. In 2025, compared with CK, the NJ9108 Si1–Si3 treatments increased NAPE, NAGE, and PFP by 17.15–34.26%, 8.84–21.72%, and 1.97–4.83%, respectively. NJ5718 increased by 17.22–39.30%, 9.71–19.29%, and 2.04–4.06%, respectively. However, NPE has shown the opposite trend over the past two years.

## 4. Discussion

### 4.1. Effect of Foliar Silicon Fertilizer on Yield

The coordination of yield components is key to achieving high rice yields [25], and the application of silicon fertilizer can significantly increase both rice yield and its component factors [26,27]. The effects of foliar silicon fertilizer application at different growth stages on yield and its components vary considerably with the yield-increasing effect of coordinated multiple stages of spray application being the most significant. Previous studies have shown that applying silicon fertilizer at the elongation and booting stages has the most significant effect on increasing rice yield [28]. The results of this paper indicate that silicon fertilizer primarily increase yield by increasing the number of panicles and spikelets per panicle, and that different application timings result in varying effects on optimizing yield components. Chu et al. [29] showed that applying silicon fertilizer whether as a basal application or foliar spray during the tillering stage effectively increased the number of tillers in rice. The results of this paper showed that treatments involving foliar silicon application at the tillering period (Si1 and Si2) had significantly higher numbers of effective panicles and percentages of productive tillers than CK and Si3 treatments, and the increase in tiller number further supports this conclusion. This is primarily because the tillering stage is a critical period for rice tiller development. After silicon fertilizer is absorbed by rice roots, it promotes root development and enhances photosynthesis, thereby providing a material basis for effective tiller and the formation of large panicles. The elongation stage coincides with the crucial phase of young panicle differentiation; thus, silicon application at this stage mainly affects the number of spikelets per panicle and carbon with nitrogen metabolism and delays spikelet degeneration. Gong et al. [19] had shown that rice accumulates significantly more silicon during the panicle initiation to heading stage than during the transplanting to panicle initiation stage, indicating that a foliar application of silicon fertilizer during the panicle initiation stage is fundamental for increasing panicles and yield. The results of this paper showed that treatments with silicon fertilizer applied at the elongation stage (Si1, Si2, Si3) had significantly higher numbers of spikelets per panicle compared with CK. Studies have shown that applying silicon fertilizer during the reproductive growth stage can regulate the expression of the silicon transporter gene Lsi6, ensuring an effective transport of silicon to large panicles and thereby supporting the formation of grain number [30]. Silicon has limited mobility within plant tissues; only a small amount of silicon absorbed by leaves during the grain-filling period is translocated into grains, and it maintains photosynthetic capacity in the later stage grain filling by delaying leaf senescence, thereby ensuring full grain development. The application of silicon fertilizer at different growth stages regulates yield components through distinct pathways. Therefore, application three times during the tillering, elongation, and grain-filling stages can comprehensively enhance all of the yield components, thereby maximizing the grain yield.

The continuous increase in dry matter accumulation is closely related to improved rice yield, as yield formation mainly depends on the production and distribution of dry matter [31]. Previous studies have shown that silicon fertilizers significantly increased plant dry matter accumulation and enhanced rice root vitality, thereby facilitating the transport of dry matter to the aboveground parts [32]. The results of this paper indicated that a foliar application of silicon fertilizer during the tillering, elongation and maturity stages enhanced dry matter accumulation by strengthening grain filling and regulating dry matter translocation, ultimately influencing yield formation. This was primarily because applying silicon fertilizer during the tillering stage promoted early plant growth, which laid a foundation for high yield. Application during the elongation stage increased stem weight, and application during the grain-filling stage extended rice photosynthesis, promoting post-flowering dry matter accumulation and translocation, and thereby increasing yield.

### 4.2. Effect of Foliar Silicon Fertilizer on Quality

Research indicated that the foliar application of silicon fertilizer can significantly increase the head milling rice rate, reduce the chalkiness degree and chalky grain percentage, enhance the taste value, and effectively improve the overall quality of rice [33,34]. Jiang et al. [15] demonstrated that silicon fertilizer improved rice quality by enhancing the activities of ADPGase, SSS, and SBE through the regulation of starch synthesis enzymes (such as AGPAI, SBEI, and SBEIIb), and it also reduced the protein content, amylose content and chalkiness degree. The results of this paper indicated that a foliar application of silicon fertilizer improved the processing, appearance, and taste qualities of rice. Overall, as the number of silicon fertilizer applications increased, rice quality showed a gradual improvement trend with three applications (Si2) demonstrating the best overall effect, which was followed by two applications (Si1, Si2).Wei et al. [35] found that silicon fertilizer primarily improved the head milling rice rate and taste value by increasing the glumes weight as well as reducing the brown rice and milling rice rates. The optimal application period was foliar application during the booting stage combined with a soil application of silicon fertilizer at the fourth leaf from the top, where increased silicon fertilizer enhances photosynthesis and dry matter accumulation. Previous studies have shown that the application of silicon fertilizer increased the head milling rice rate from Si0 (the application rate of silicon fertilizer is 0 kg hm^−2^) to Si400 (the application rate of silicon fertilizer is 400 kg hm^−2^) treatment, while the broken rice rate decreased by 19% and chalkiness decreased by 55%; at that time, the taste value also improved [36]. The results of this paper showed that with silicon fertilizer application, the head milling rice rate in Si2 treatment was the highest across both varieties and two years, significantly outperforming Si3 and Si1. This may be due to substantial silicon deposition in the glumes: three applications enhanced the accumulation effect of silicon, reduced rice breakage during milling and thereby increased the head milling rice rate. In terms of appearance quality, the chalky grain percentage and chalkiness degree showed a decreasing trend. The chalkiness degree of Si2 treatment was the lowest among both varieties in two years of trials. In 2024, compared with CK, the Si2 treatment reduced chalkiness degree by 5.45% and 3.42% for NJ9108 and NJ5718, respectively. This may be due to silicon promoting the transport of filling substances into grains and effectively reducing the gaps between endosperm cells, further reducing chalkiness. Moreover, Si3 and Si2 treatments showed better performance in taste value compared to Si1 and CK, which was primarily because silicon fertilizer applied during the grain-filling stage directly participates in grain metabolism, more effectively regulating starch synthase activity and related gene expression. This targeted regulation particularly reduces amylose and protein content, resulting in superior eating quality for Si2 and Si3.

### 4.3. Effect of Foliar Silicon Fertilizer on Lodging Resistance and N Use Efficiency

Lodging is one of the key factors threatening high and stable rice yields [37]. Silicon plays a crucial role in enhancing plant uprightness and mechanical strength. Applying silicon fertilizer during stem development can strengthen stem mechanical resistance and improve plant lodging tolerance [38,39]. Plant architecture plays a decisive role in lodging resistance, particularly the length and wall thickness of the basal internodes [40]. Studies have shown that silicon fertilizer can increase the lignin and cellulose content in stems. Silicon deposits into vascular bundle cells, combining with cellulose and hemicellulose to form new complexes, which promote the differentiation and thickening of plant thick-walled tissues. Applying silicon fertilizer during the elongation and heading stages significantly increased the silicon content in stems as well as the lignin and cellulose levels in cell walls. This indirectly confirmed that applying silicon fertilizer during the reproductive growth period was far more effective than during the grain-filling stage in promoting stem structural development and enhancing stem strength [41]. The results of this paper showed that there were no significant differences in the bending resistance and stem wall thickness between Si2 treatment and Si1 or Si3 treatments, which further indicated that the elongation stage was the key period for applying silicon fertilizer to enhance rice lodging resistance. Comparisons among different silicon fertilizer treatments revealed that during the tillering stage, rice stems are in the early differentiation phase, and applying silicon fertilizer at this stage lays a foundation for developing thicker stems later on. In contrast, by the grain-filling stage, stem growth has largely ceased, and no significant changes in internode length or stem wall thickness occur. Therefore, applying silicon fertilizer during both the tillering and elongation stages can effectively improve rice’s lodging resistance. The second and third internodes at the base are the primary sites bearing bending moments. Under Si2 treatment, both NJ9108 and NJ5718 showed stem diameter increases of over 30% in the second and third basal internodes compared with CK along with a significant reduction in internode length. The combined effect of reduced length and increased stem diameter significantly enhanced bending resistance, which is consistent with previous studies.

Previous studies have shown that nitrogen accumulation in rice is significantly influenced by silicon accumulation, and silicon fertilizer can enhance nitrogen uptake in rice [42]. Berahim et al. [43] demonstrated that the foliar application of silicon fertilizer increased the accumulation of nitrogen aboveground at the maturity stage. The results of this paper showed that as the growth stage progressed, nitrogen accumulation in rice gradually increased, reaching its peak at the maturity stage. Silicon enhances root activity and promotes the uptake of ammonium and nitrate nitrogen from soil [44]. In treatments with silicon fertilizer application, nitrogen accumulation at the elongation, heading, and maturity stages was significantly higher than of the control (CK), indicating that silicon has a cumulative effect on nitrogen uptake. The significant increase in NAPE and NAGE indicates that under the same nitrogen application rate (270 kg ha^−1^), applying silicon fertilizer can improve nitrogen use efficiency, thereby increasing nitrogen accumulation in plants. The declining trend in NPE may be due to silicon promoting rapid nitrogen uptake, but part of the nitrogen is temporarily stored in nutrient organs such as stems and leaves rather than being fully and efficiently converted into grain yield, resulting in a lower nitrogen use efficiency per unit of nitrogen applied. Nevertheless, the overall grain yield still increases.

## 5. Conclusions

The foliar application of silicon fertilizer significantly improved the rice yield, quality, lodging resistance, and nitrogen use efficiency, but the regulatory effects vary depended on the application timing. Two applications during the tillering and elongation stages (Si1) significantly increased the number of effective panicles and spikelets per panicle in rice, thereby enhancing the dry matter accumulation and stem lodging resistance of the basal internodes, but it had a limited effect on delaying leaf senescence in later growth stages. Although the foliar application of silicon (Si3) at the elongation and grain-filling stages significantly improved taste quality and effectively delayed leaf senescence, the lack of silicon application at the tillering stage resulted in a smaller increase in the number of panicles compared with treatments Si1 and Si2. Overall, the combined application at the tillering, elongation and grain-filling stages (Si2) achieved the optimal performance: it not only significantly increased the panicles and spikelets per panicle but also delayed leaf senescence in rice, ultimately increasing rice yield by 4.02–5.70%. Meanwhile, Si2 thickened the basal internodes of the rice stem (by more than 30%) and reduced the length of basal internodes, thereby improving the lodging resistance. The Si2 treatment significantly increased the head-grain milling rate and reduced the chalkiness degree. Silicon fertilizer was applied at the grain-filling stage in Si2 and Si3 treatments, which regulated the activity of starch synthases and consequently enhanced the taste value of rice. Additionally, nitrogen accumulation was significantly higher than that of the control (CK) following silicon applications at the elongation, heading, and maturity stages. Nitrogen use efficiency increased with the delay of silicon fertilizer application time; compared with silicon application at the tillering stage, application at the grain-filling stage was more conducive to nitrogen transport and utilization. Overall, three consecutive applications at the tillering, elongation, and grain-filling stages can optimize the yield, quality, lodging resistance and nitrogen use efficiency of japonica rice: preliminary estimates indicated that the cost of a single foliar application of silicon fertilizer is significantly lower than the increase in output value resulting from the yield increase, demonstrating that this measure has good economic feasibility under the experimental conditions, and providing both theoretical and technical support for high-yield and high-quality japonica rice cultivation.

## Figures and Tables

**Table 1:** Treatment numbers and timing of silica fertilizer application.

Treatment	Spraying Period
CK	-
Si1	tillering + elongation stages
Si2	tillering + elongation + grain-filling stages
Si3	elongation + grain-filling stages

**Table 2:** Effects of foliar silicon fertilizer on rice yield and yield components.

Year	Cultivars	Treatment	Panicles (×10^4^·hm^−2^)	Spikelets per Panicle	Seed-Setting Rate (%)	1000-Grain Weight (g)
2024	NJ9108	CK	305.78 ^c^	141.34 ^c^	91.75 ^a^	24.69 ^a^
		Si1	315.46 ^ab^	145.89 ^ab^	91.87 ^a^	24.64 ^a^
		Si2	316.09 ^a^	146.16 ^a^	92.01 ^a^	24.60 ^a^
		Si3	313.31 ^b^	145.46 ^b^	91.95 ^a^	24.75 ^a^
	NJ5718	CK	356.67 ^d^	145.14 ^c^	92.75 ^a^	24.95 ^a^
		Si1	362.52 ^b^	149.79 ^ab^	92.70 ^a^	24.97 ^a^
		Si2	363.19 ^a^	150.36 ^a^	92.81 ^a^	25.01 ^a^
		Si3	360.47 ^c^	148.72 ^b^	92.76 ^a^	24.98 ^a^
2025	NJ9108	CK	308.71 ^c^	145.11 ^c^	91.25 ^a^	25.34 ^a^
		Si1	317.16 ^ab^	147.81 ^ab^	91.37 ^a^	25.32 ^a^
		Si2	317.55 ^a^	148.02 ^a^	91.51 ^a^	25.43 ^a^
		Si3	315.95 ^b^	147.45 ^b^	91.45 ^a^	25.37 ^a^
	NJ5718	CK	355.67 ^c^	142.08 ^c^	92.30 ^a^	25.22 ^a^
		Si1	362.52 ^a^	146.87 ^ab^	92.22 ^a^	25.30 ^a^
		Si2	363.19 ^a^	147.18 ^a^	92.35 ^a^	25.28 ^a^
		Si3	360.47 ^b^	145.78 ^b^	92.25 ^a^	25.16 ^a^

Different lowercase letters following data within the same column indicate significant differences among treatments at the 5% probability level (*p* < 0.05). The same convention applies to subsequent tables.

**Table 3:** Effects of foliar silicon fertilizer on leaf area index.

Year	Cultivars	Treatment	LAI			Decreasing Rate of Leaf Area (LAI·d^−1^)
			Elongation	Heading	Maturity	
2024	NJ9108	CK	3.48 ^b^	7.20 ^c^	3.59 ^c^	0.0694 ^a^
		Si1	3.81 ^a^	7.49 ^a^	3.92 ^b^	0.0686 ^ab^
		Si2	3.83 ^a^	7.52 ^a^	4.14 ^a^	0.0650 ^c^
		Si3	3.47 ^b^	7.38 ^b^	3.96 ^b^	0.0658 ^bc^
	NJ5718	CK	4.06 ^b^	7.45 ^c^	3.25 ^c^	0.0808 ^a^
		Si1	4.18 ^a^	7.77 ^ab^	3.58 ^b^	0.0806 ^ab^
		Si2	4.17 ^a^	7.80 ^a^	3.81 ^a^	0.0766 ^b^
		Si3	4.03 ^b^	7.67 ^b^	3.65 ^ab^	0.0774 ^ab^
2025	NJ9108	CK	3.65 ^b^	7.33 ^c^	3.53 ^c^	0.0761 ^a^
		Si1	3.97 ^a^	7.70 ^a^	3.91 ^b^	0.0752 ^ab^
		Si2	3.95 ^a^	7.69 ^a^	4.07 ^a^	0.0724 ^c^
		Si3	3.63 ^b^	7.59 ^b^	3.94 ^b^	0.0736 ^bc^
	NJ5718	CK	4.09 ^b^	7.29 ^c^	3.2 ^c^	0.0802 ^a^
		Si1	4.21 ^a^	7.65 ^a^	3.67 ^b^	0.0796 ^a^
		Si2	4.19 ± 0.01 ^a^	7.64 ^a^	3.80 ^a^	0.0768 ^b^
		Si3	4.11 ^b^	7.59 ^b^	3.74 ^ab^	0.0770 ^b^

Different lowercase letters following data within the same column indicate significant differences among treatments at the 5% probability level (*p* < 0.05). The same convention applies to subsequent tables.

**Table 4:** Effects of foliar silicon fertilizer on tiller dynamics.

Year	Cultivars	Treatment	Tiller Number (×10^4^ hm^−2^)			Percentage of Productive Tillers (%)
			Elongation	Heading	Maturity	
2024	NJ9108	CK	448.36 ^b^	402.03 ^c^	316.40 ^d^	70.57 ^c^
		Si1	472.76 ^a^	433.33 ^a^	342.62 ^b^	72.48 ^ab^
		Si2	474.74 ^a^	434.33 ^a^	349.41 ^a^	73.61 ^a^
		Si3	450.88 ^b^	413.35 ^b^	323.16 ^c^	71.67 ^bc^
	NJ5718	CK	450.32 ^b^	408.70 ^c^	334.86 ^d^	74.37 ^b^
		Si1	460.39 ^a^	420.48 ^a^	344.74 ^b^	74.88 ^b^
		Si2	459.78 ^a^	419.60 ^a^	353.87 ^a^	76.96 ^a^
		Si3	450.47 ^b^	413.87 ^b^	339.35 ^c^	75.33 ^b^
2025	NJ9108	CK	446.67 ^b^	405.67 ^c^	318.11 ^d^	71.22 ^b^
		Si1	475.67 ^a^	437.23 ^a^	343.15 ^b^	72.14 ^ab^
		Si2	477.64 ^a^	439.19 ^a^	348.45 ^a^	72.95 ^a^
		Si3	448.55 ^b^	417.09 ^b^	322.72 ^c^	71.95 ^b^
	NJ5718	CK	456.02 ^b^	414.63 ^c^	335.29 ^c^	73.53 ^c^
		Si1	462.91 ^a^	423.43 ^a^	345.12 ^b^	74.56 ^bc^
		Si2	462.35 ^a^	424.45 ^a^	350.88 ^a^	75.89 ^a^
		Si3	455.78 ^b^	418.80 ^b^	340.86 ^b^	74.79 ^ab^

Different lowercase letters following data within the same column indicate significant differences among treatments at the 5% probability level (*p* < 0.05). The same convention applies to subsequent tables.

**Table 5:** Effects of foliar silicon fertilizer on dry matter accumulation.

Year	Cultivars	Treatment	Dry Matter Accumulation (t hm^−2^)		
			Elongation	Heading	Maturity
2024	NJ9108	CK	5.29 ^b^	13.07 ^c^	21.05 ^d^
		Si1	5.47 ^a^	13.49 ^a^	21.64 ^c^
		Si2	5.49 ^a^	13.51 ^a^	22.17 ^a^
		Si3	5.31 ^b^	13.26 ^b^	21.83 ^b^
	NJ5718	CK	5.07 ^b^	12.15 ^c^	20.76 ^c^
		Si1	5.36 ^a^	12.53 ^a^	21.14 ^b^
		Si2	5.38 ^a^	12.50 ^ab^	21.46 ^a^
		Si3	5.08 ^b^	12.32 ^bc^	21.34 ^a^
2025	NJ9108	CK	5.30 ^b^	13.04 ^c^	21.66 ^c^
		Si1	5.55 ^a^	13.50 ^a^	22.45 ^b^
		Si2	5.53 ^a^	13.51 ^a^	22.74 ^a^
		Si3	5.31 ^b^	13.23 ^b^	22.67 ^a^
	NJ5718	CK	5.14 ^b^	12.16 ^c^	21.17 ^d^
		Si1	5.47 ^a^	12.57 ^a^	21.64 ^c^
		Si2	5.49 ^a^	12.58 ^a^	21.84 ^a^
		Si3	5.16 ^b^	12.34 ^b^	21.74 ^b^

Different lowercase letters following data within the same column indicate significant differences among treatments at the 5% probability level (*p* < 0.05). The same convention applies to subsequent tables.

**Table 6:** Effects of foliar silicon fertilizer on processing and appearance quality.

Year	Cultivars	Treatment	Brown Rice Rate (%)	Milling Rice Rate (%)	Head Milling Rate (%)	Chalky Grain Percentage (%)	Chalkiness Degree (%)
2024	NJ9108	CK	81.07 ^c^	71.06 ^c^	61.63 ^c^	41.90 ^a^	11.93 ^a^
		Si1	82.18 ^b^	71.35 ^b^	62.11 ^b^	40.27 ^b^	11.58 ^a^
		Si2	82.74 ^a^	71.53 ^a^	62.52 ^a^	38.44 ^d^	11.28 ^a^
		Si3	82.52 ^ab^	71.41 ^b^	62.40 ^ab^	38.85 ^c^	11.36 ^a^
	NJ5718	CK	80.96 ^b^	69.57 ^c^	51.97 ^c^	38.93 ^a^	20.49 ^a^
		Si1	81.49 ^a^	69.95 ^b^	52.49 ^b^	38.14 ^b^	20.06 ^a^
		Si2	81.62 ^a^	70.13 ^a^	52.76 ^a^	36.18 ^d^	19.79 ^a^
		Si3	81.56 ^a^	70.10 ^a^	52.62 ^ab^	37.13 ^c^	19.87 ^a^
2025	NJ9108	CK	83.60 ^c^	71.68 ^c^	61.99 ^c^	42.14 ^a^	11.82 ^a^
		Si1	83.83 ^b^	71.96 ^b^	62.60 ^b^	40.58± ^b^	11.46 ^a^
		Si2	84.04 ^a^	72.14 ^a^	63.02 ^a^	38.73 ^d^	11.15 ^a^
		Si3	83.97 ^ab^	72.02 ^b^	62.90 ^a^	39.14 ^c^	11.23 ^a^
	NJ5718	CK	82.43 ^b^	70.95 ^c^	53.21 ^c^	40.07 ^a^	21.47 ^a^
		Si1	82.98 ^a^	71.34 ^b^	53.73 ^b^	39.26 ^b^	21.04 ^a^
		Si2	83.11 ^a^	71.52 ^a^	54.01 ^a^	37.29 ^d^	20.77 ^a^
		Si3	83.05 ^a^	71.49 ^a^	53.88 ^ab^	38.25 ^c^	20.84 ^a^

Different lowercase letters following data within the same column indicate significant differences among treatments at the 5% probability level (*p* < 0.05). The same convention applies to subsequent tables.

**Table 7:** Effects of foliar silicon fertilizer on nutritional and taste quality.

Year	Cultivars	Treatment	Protein Content (%)	Amylose Content (%)	Taste Value
2024	NJ9108	CK	7.20 ^a^	10.30 ^a^	76.32 ^c^
		Si1	7.05 ^ab^	10.19 ^a^	78.45 ^b^
		Si2	6.80 ^b^	9.75 ^b^	80.29 ^a^
		Si3	6.86 ^b^	9.81 ^b^	80.81 ^a^
	NJ5718	CK	7.50 ^a^	10.66 ^a^	77.72 ^b^
		Si1	7.27 ^b^	10.53 ^b^	78.21 ^ab^
		Si2	7.08 ^c^	10.16 ^c^	78.77 ^a^
		Si3	7.03 ^c^	10.20 ^c^	79.07 ^a^
2025	NJ9108	CK	7.36 ^a^	10.24 ^a^	77.20 ^c^
		Si1	7.24 ^ab^	10.16 ^ab^	79.34 ^b^
		Si2	7.12 ^b^	9.95 ^b^	81.18 ^a^
		Si3	7.09 ^b^	9.89 ^b^	81.71 ^a^
	NJ5718	CK	7.99 ^a^	10.49 ^a^	79.56 ^c^
		Si1	7.72 ^ab^	10.38 ^ab^	80.95 ^b^
		Si2	7.50 ^b^	10.24 ^b^	81.15 ^a^
		Si3	7.44 ^b^	10.17 ^b^	81.24 ^a^

Different lowercase letters following data within the same column indicate significant differences among treatments at the 5% probability level (*p* < 0.05). The same convention applies to subsequent tables.

**Table 8:** Effect of foliar silicon fertilizer on bending resistance and stem wall thickness.

Year	Cultivars	Treatment	N2 Bending Resistance (N)	N3 Bending Resistance (N)	N2 Stem Wall Thickness (mm)	N3 Stem Wall Thickness (mm)
2024	NJ9108	CK	13.47 ^c^	8.06 ^b^	0.80 ^b^	0.67 ^b^
		Si1	14.46 ^ab^	8.62 ^a^	0.82 ^ab^	0.73 ^ab^
		Si2	14.56 ^a^	8.64 ^a^	0.88 ^a^	0.75 ^a^
		Si3	14.18 ^b^	8.45 ^a^	0.85 ^ab^	0.73 ^ab^
	NJ5718	CK	15.39 ^c^	11.23 ^b^	0.94 ^b^	0.78 ^b^
		Si1	16.04 ^ab^	12.17 ^a^	1.05 ^a^	0.85 ^ab^
		Si2	16.12 ^a^	12.23 ^a^	1.08 ^a^	0.90 ^a^
		Si3	15.83 ^b^	12.11 ^a^	1.03 ^a^	0.89 ^a^
2025	NJ9108	CK	13.69 ^c^	8.35 ^c^	0.85 ^b^	0.72 ^b^
		Si1	14.70 ^ab^	8.87 ^ab^	0.88 ^ab^	0.77 ^a^
		Si2	14.77 ^a^	8.94 ^a^	0.93 ^a^	0.79 ^a^
		Si3	14.48 ^b^	8.75 ^b^	0.89 ^ab^	0.78 ^a^
	NJ5718	CK	15.75 ^c^	11.94 ^b^	1.09 ^b^	0.83 ^b^
		Si1	16.49 ^a^	12.41 ^a^	1.14 ^a^	0.91 ^a^
		Si2	16.57 ^a^	12.49 ^a^	1.15 ^a^	0.92 ^a^
		Si3	16.38 ^b^	12.35 ^a^	1.13 ^a^	0.90 ^a^

Different lowercase letters following data within the same column indicate significant differences among treatments at the 5% probability level (*p* < 0.05). The same convention applies to subsequent tables.

**Table 9:** Effect of foliar silicon fertilizer on N accumulation.

Year	Cultivars	Treatment	N Accumulation (kg hm^−2^)		
			Elongation	Heading	Maturity
2024	NJ9108	CK	94.06 ^b^	164.28 ^c^	233.66 ^c^
		Si1	110.56 ^a^	191.06 ^a^	248.12 ^b^
		Si2	108.24 ^a^	190.54 ^a^	265.29 ^a^
		Si3	97.64 ^b^	175.46 ^b^	256.18 ^ab^
	NJ5718	CK	97.12 ^b^	169.68 ^c^	245.01 ^d^
		Si1	111.66 ^a^	196.31 ^a^	258.17 ^c^
		Si2	112.54 ^a^	198.39 ^a^	278.98 ^a^
		Si3	98.72 ^b^	179.93 ^b^	267.41 ^b^
2025	NJ9108	CK	95.93 ^b^	167.35 ^c^	236.78 ^c^
		Si1	112.66 ^a^	191.25 ^a^	252.18 ^b^
		Si2	111.34 ^a^	193.20 ^a^	267.60 ^a^
		Si3	98.75 ^b^	179.93 ^b^	259.18 ^ab^
	NJ5718	CK	98.51 ^b^	173.88 ^c^	249.81 ^d^
		Si1	114.03 ^a^	199.08 ^a^	264.73 ^c^
		Si2	114.66 ^a^	200.40 ^a^	283.89 ^a^
		Si3	100.21 ^b^	180.99 ^b^	272.47 ^b^

Different lowercase letters following data within the same column indicate significant differences among treatments at the 5% probability level (*p* < 0.05). The same convention applies to subsequent tables.

**Table 10:** Effects of foliar silicon fertilizer on nitrogen use efficiency.

Year	Cultivars	Treatment	NAPE (%)	NAGE (kg·kg^−1^)	NPE (kg·kg^−1^)	PFP (kg·kg^−1^)
2024	NJ9108	CK	32.85 ^c^	8.34 ^d^	25.45 ^a^	33.16 ^d^
		Si1	38.20 ^b^	9.21 ^c^	24.16 ^ab^	34.02 ^c^
		Si2	44.56 ^a^	10.22 ^a^	22.97 ^b^	35.04 ^a^
		Si3	41.18 ^ab^	9.66 ^b^	23.45 ^ab^	34.48 ^b^
	NJ5718	CK	29.82 ^d^	8.34 ^d^	28.01 ^a^	37.07 ^d^
		Si1	34.69 ^c^	9.38 ^c^	27.07 ^ab^	38.11 ^c^
		Si2	42.39 ^a^	10.33 ^a^	24.36 ^c^	39.05 ^a^
		Si3	38.11 ^b^	9.84 ^b^	25.81 ^b^	38.57 ^b^
2025	NJ9108	CK	33.30 ^c^	7.69 ^d^	23.13 ^a^	34.58 ^d^
		Si1	39.01 ^b^	8.37 ^c^	21.47 ^b^	35.26 ^c^
		Si2	44.71 ^a^	9.36 ^a^	20.95 ^b^	36.25 ^a^
		Si3	41.60 ^ab^	8.84 ^b^	21.29 ^b^	35.73 ^b^
	NJ5718	CK	32.11 ^d^	8.14 ^c^	25.44 ^a^	38.69 ^c^
		Si1	37.64 ^c^	8.93 ^b^	23.77 ^ab^	39.48 ^b^
		Si2	44.73 ^a^	9.71 ^a^	21.70 ^b^	40.26 ^a^
		Si3	40.51 ^b^	9.21 ^b^	22.84 ^b^	39.76 ^b^

Different lowercase letters following data within the same column indicate significant differences among treatments at the 5% probability level (*p* < 0.05). The same convention applies to subsequent tables.

## Data Availability

The original contributions presented in this study are included in the article. Further inquiries can be directed to the corresponding authors.

## References

[1] Liao P., Bell S.M., Chen L., Huang S., Wang H., Miao J., Qi Y., Sun Y., Liao B., Zeng Y. (2023). Improving Rice Grain Yield and Reducing Lodging Risk Simultaneously: A Meta-Analysis. Eur. J. Agron..

[2] Luo Z., Liang X., Lam S.K., Mosier A.R., Hu S., Chen D. (2021). Hotspots of Reactive Nitrogen Loss in China: Production, Consumption, Spatiotemporal Trend and Reduction Responsibility. Environ. Pollut..

[3] Salassi M.E., Deliberto M.A., Linscombe S.D., Wilson C.E., Walker T.W., McCauley G.N., Blouin D.C. (2013). Impact of Harvest Lodging on Rough Rice Milling Yield and Market Price. Agron. J..

[4] Berry P.M., Sterling M., Spink J.H., Baker C.J., Sylvester-Bradley R., Mooney S.J., Tams A.R., Ennos A.R. (2004). Understanding and Reducing Lodging in Cereals. Advances in Agronomy.

[5] Chen Y., Liu Y., Dong S., Liu J., Wang Y., Hussain S., Wei H., Huo Z., Xu K., Dai Q. (2022). Response of Rice Yield and Grain Quality to Combined Nitrogen Application Rate and Planting Density in Saline Area. Agriculture.

[6] Alvarez J., Datnoff L.E. (2001). The Economic Potential of Silicon for Integrated Management and Sustainable Rice Production. Crop Prot..

[7] Chang Y., Cui H., Wang Y., Li C., Wang J., Jin M., Luo Y., Li Y., Wang Z. (2023). Silicon Spraying Enhances Wheat Stem Resistance to Lodging under Light Stress. Agronomy.

[8] Ma J.F., Tamai K., Yamaji N., Mitani N., Konishi S., Katsuhara M., Ishiguro M., Murata Y., Yano M. (2006). A Silicon Transporter in Rice. Nature.

[9] Ma J.F., Yamaji N. (2006). Silicon Uptake and Accumulation in Higher Plants. Trends Plant Sci..

[10] Gong D., Zhang X., Yao J., Dai G., Yu G., Zhu Q., Gao Q., Zheng W. (2021). Synergistic Effects of Bast Fiber Seedling Film and Nano-Silicon Fertilizer to Increase the Lodging Resistance and Yield of Rice. Sci. Rep..

[11] Jadhao K.R., Rout G.R. (2020). Silicon (Si) Enhances the Resistance in Finger Millet Genotypes against Blast Disease. J. Plant Pathol..

[12] Bhardwaj S., Kapoor D. (2021). Fascinating Regulatory Mechanism of Silicon for Alleviating Drought Stress in Plants. Plant Physiol. Biochem..

[13] Hou L., Ji S., Zhang Y., Wu X., Zhang L., Liu P. (2023). The Mechanism of Silicon on Alleviating Cadmium Toxicity in Plants: A Review. Front. Plant Sci..

[14] Wei X., Wu G., Wang B., Jiang Z., Wei Z., Wang S., Song H., Tian P., Wu Z., Yang M. (2025). Optimized Application Strategy of Nitrogen–Silicon (N-Si) Fertilization Improves Nitrogen Metabolism, Grain Yield, and Growth of Rice under Dry Cultivation. BMC Plant Biol..

[15] Jiang H., Xu X., Sun A., Bai C., Li Y., Nuo M., Shen X., Li W., Wang D., Tian P. (2024). Silicon Nutrition Improves the Quality and Yield of Rice under Dry Cultivation. J. Sci. Food Agric..

[16] Thorne S.J., Maathuis F.J.M., Hartley S.E. (2023). Induction of Silicon Defences in Wheat Landraces Is Local, Not Systemic, and Driven by Mobilization of Soluble Silicon to Damaged Leaves. J. Exp. Bot..

[17] Maghsoudi K., Emam Y., Ashraf M. (2016). Foliar Application of Silicon at Different Growth Stages Alters Growth and Yield of Selected Wheat Cultivars. J. Plant Nutr..

[18] Velasco E., Aranda X., Houben F., Ribes J., Araus J.L., García-Caparros P. (2026). Interactive Effects of Silicon Formulations, Concentrations, and Foliar Application Timing on Rice Physiology and Yield. Front. Plant Sci..

[19] Gong J., Hu Y., Long H., Chang Y., Ge M., Gao H., Liu Y., Zhang H., Dai Q., Huo Z. (2012). Effect of Application of Silicon at Different Periods on Grain Yield and Silicon Absorption, Use Efficiency in Super Rice. Sci. Agric. Sin..

[20] Dorairaj D., Ismail M.R., Sinniah U.R., Tan K.B. (2020). Silicon Mediated Improvement in Agronomic Traits, Physiological Parameters and Fiber Content in *Oryza sativa*. Acta Physiol. Plant.

[21] Xu Z., Li T., Cui J., Yu J., Li G., Zhu Y., Liu G., Xu F., Hu Q., Wei H. (2026). Effects of Nitrogen Application Rates and Nitrogen Topdressing at Different Leaf Growth Stages on the Yield, Nitrogen Absorption, and Utilization of Nanjing 9108. Plants.

[22] Mi K., Yuan X., Wang Q., Dun C., Wang R., Yang S., Yang Y., Zhang H., Zhang H. (2023). Zinc Oxide Nanoparticles Enhanced Rice Yield, Quality, and Zinc Content of Edible Grain Fraction Synergistically. Front. Plant Sci..

[23] Wu H., Jing W., Zhang Y., Zhang Y., Wang W., Zhu K., Zhang W., Gu J., Liu L., Zhang J. (2026). Optimized Application of Controlled-Release Nitrogen Improves Grain Yield, Nitrogen Use Efficiency, and Lodging Resistance in Rice. J. Integr. Agric..

[24] Soulaimani A., Gharous M.E., Mejahed K.E., Oulfakir R., Fathallah S., Gmouh S. (2025). Validation of Continuous Flow Analysis for Determining Total Nitrogen in Plants Compared to the Kjeldahl Method. Discov. Plants.

[25] Fu W., Zhao Y., Zha X., Ullah J., Ye M., Shah F., Yuan Q., Wang P., Tao Y., Wu W. (2023). The Potential Role of Zinc and Silicon in Improving Grain Yield and Lodging Resistance of Rice (*Oryza sativa* L.). Agronomy.

[26] Cuong T.X., Ullah H., Datta A., Hanh T.C. (2017). Effects of Silicon-Based Fertilizer on Growth, Yield and Nutrient Uptake of Rice in Tropical Zone of Vietnam. Rice Sci..

[27] Flores R.A., Pessoa-de-Souza M.A., De Andrade A.F., Bueno A.M., De Oliveira Abdala K., De Souza Júnior J.P., De Mello Prado R., Santos G.G., Mesquita M. (2022). Does Foliar Application of Silicon under Natural Water Stress Conditions Increase Rice Yield in Subtropical Dry Regions?. Silicon.

[28] Arthi V., Sriramachandrasekharan M.V., Senthilvalavan P., Manivannan R. (2023). Assessing the Impact of Co-Fertilization of Silicon with Macronutrient Fertilizers on Yield, Nutrient Uptake, Use Efficiency and Grain Quality of Rice in Sandy Clay Loam Soil. IJPSS.

[29] Chu M., Liu M., Ding Y., Wang S., Liu Z., Tang S., Ding C., Chen L., Li G. (2018). Effect of Nitrogen and Silicon on Rice Submerged at Tillering Stage. Agron. J..

[30] Chaiwong N., Pusadee T., Jamjod S., Prom-u-thai C. (2022). Silicon Application Promotes Productivity, Silicon Accumulation and Upregulates Silicon Transporter Gene Expression in Rice. Plants.

[31] He X., Zhu H., Shi A., Wang X. (2024). Optimizing Nitrogen Fertilizer Management Enhances Rice Yield, Dry Matter, and Nitrogen Use Efficiency. Agronomy.

[32] Swe M.M., Mar S.S., Naing T.T., Zar T., Ngwe K. (2021). Effect of Silicon Application on Growth, Yield and Uptake of Rice (*Oryza sativa* L.) in Two Different Soils. Open Access Libr. J..

[33] Luo H., Duan M., Xing P., Zhang Y., Qi J., Kong L., Tang X. (2023). Effects of L-Glutamic Acid Application on Yield, Grain Quality, Photosynthetic Pigments, 2-Acetyl-1-Pyrroline, and Antioxidant System of Aromatic Rice. Field Crops Res..

[34] Liu X., Huang Z., Li Y., Xie W., Li W., Tang X., Ashraf U., Kong L., Wu L., Wang S. (2020). Selenium-Silicon (Se-Si) Induced Modulations in Physio-Biochemical Responses, Grain Yield, Quality, Aroma Formation and Lodging in Fragrant Rice. Ecotoxicol. Environ. Saf..

[35] Wei X., Zhang Y., Song X., Zhao L., Zhao Q., Chen T., Lu K., Zhu Z., Huang S., Wang C. (2024). Silicon and Zinc Fertilizer Application Improves Grain Quality and Aroma in the Japonica Rice Variety Nanjing 46. Foods.

[36] Misran A. (2026). Application of Silicon Improved Rice Productivity and Reduced Chalky Rice. Asia-Pac. J. Sci. Technol..

[37] Liu W., Gai D., Liang J., Cui J., Geng Y., Zhang Q., Guo L., Shao X. (2025). Paclobutrazol Enhances Lodging Resistance and Yield of Direct-Seeded Rice by Optimizing Plant Type and Canopy Light Transmittance. Field Crops Res..

[38] Zhou T., Cui R., Shu C., Zhu K., Zhang W., Zhang H., Liu L., Wang Z., Gu J., Yang J. (2024). Combining Urea and Controlled Release Nitrogen Fertilizer to Enhance Lodging Resistance of Rice (*Oryza sativa* L.) by Altering Accumulation of Silicon and Cell Wall Polymers at High Yielding Levels. Field Crops Res..

[39] Hong W., Chen Y., Huang S., Li Y., Wang Z., Tang X., Pan S., Tian H., Mo Z. (2022). Optimization of Nitrogen–Silicon (N-Si) Fertilization for Grain Yield and Lodging Resistance of Early-Season Indica Fragrant Rice under Different Planting Methods. Eur. J. Agron..

[40] Wu M., Li L., Wu G., Meng X., Wang Z., Zhang H., Xue B., Liu Z., Li H., Liu Z. (2025). Applying Silicon Fertilizer under Straw Return Can Reduce Nitrogen Application, Increase Rice Yield and Lodging Resistance. BMC Plant Biol..

[41] Ao D., Li Z., Dou Z., Liao P., Song J., Dai Q., Gao H., Xu Q. (2025). Optimizing Deep Nitrogen Placement and Silicon Fertilizer Use for Enhanced Rice Productivity and Lodging Resistance Under Different Irrigation Modes. Food Energy Secur..

[42] Zhang S., Yang Y., Zhai W., Tong Z., Shen T., Li Y.C., Zhang M., Sigua G.C., Chen J., Ding F. (2019). Controlled-Release Nitrogen Fertilizer Improved Lodging Resistance and Potassium and Silicon Uptake of Direct-Seeded Rice. Crop Sci..

[43] Berahim Z., Omar M.H., Zakaria N.-I., Ismail M.R., Rosle R., Roslin N.A., Che’Ya N.N. (2021). Silicon Improves Yield Performance by Enhancement in Physiological Responses, Crop Imagery, and Leaf and Culm Sheath Morphology in New Rice Line, PadiU Putra. BioMed Res. Int..

[44] Pavlovic J., Kostic L., Bosnic P., Kirkby E.A., Nikolic M. (2021). Interactions of Silicon with Essential and Beneficial Elements in Plants. Front. Plant Sci..

